# Tinnitus reduction in patients with single-sided deafness: the effect of cochlear implantation, bone conduction devices, and contralateral routing of sound hearing aids investigated in a randomized controlled trial

**DOI:** 10.3389/fneur.2024.1428106

**Published:** 2024-07-23

**Authors:** Anne W. Wendrich, Kelly K. S. Assouly, Jan A. A. van Heteren, Jeroen P. M. Peters, Wilko Grolman, Robert J. Stokroos, Huib Versnel, Adriana L. Smit

**Affiliations:** ^1^Department of Otorhinolaryngology, Head and Neck Surgery, University Medical Center Utrecht, Utrecht University, Utrecht, Netherlands; ^2^UMC Utrecht Brain Center, University Medical Center Utrecht, Utrecht, Netherlands; ^3^Cochlear Technology Centre, Mechelen, Belgium; ^4^Jean Causse Ear Clinic, Traverse de Béziers, Colombiers, France

**Keywords:** randomized controlled trial, single-sided deafness, bone conduction device, contralateral routing of sound, tinnitus

## Abstract

**Objectives:**

Single-sided deafness (SSD) is often accompanied by tinnitus, resulting in a decreased quality of life. Currently, there is a lack of high level of evidence studies comparing different treatment options for SSD regarding tinnitus reduction. This randomized controlled trial (RCT) evaluated the effect of a cochlear implant (CI), bone conduction device (BCD), contralateral routing of sound (CROS), and no treatment on tinnitus outcomes in SSD patients, with follow-up extending to 24 months.

**Methods:**

A total of 120 adult SSD patients were randomized to three groups: CI, a trial period with first a BCD on a headband, then a CROS, or vice versa. After the trial periods, patients opted for a BCD, CROS, or no treatment. At the start of follow-up, 28 patients were implanted with a CI, 25 patients with a BCD, 34 patients had a CROS, and 26 patients chose no treatment. The Tinnitus Handicap Inventory (THI), Tinnitus Questionnaire (TQ), the Visual Analog Scale (VAS), and the Hospital Anxiety and Depression Scale (HADS) were completed at baseline and at 3, 6, 12, and 24 months of follow-up.

**Results:**

The CI and BCD groups showed significantly decreased tinnitus impact scores. The CI group showed the largest decrease, which was already observed at 3 months of follow-up. Compared to the baseline, the median THI score decreased by 23 points, the TQ score by 17 points, and the VAS score by 60 points at 24 months. In the BCD group, the TQ score decreased by 9 points, and the VAS decreased by 25 points at 24 months. The HADS anxiety and depression subscale showed no indication for anxiety or depression at baseline, nor at 24 months, for all groups.

**Conclusion:**

In this RCT, SSD patients treated with a CI or BCD showed an overall decrease in tinnitus impact scores up to 24 months compared to baseline. The CI group reported a stable and the largest reduction. Cochlear implants appear to be superior to BCD and CROS, and no treatment for achieving partial or complete resolution of tinnitus in patients with SSD.

**Clinical trial registration:**

Netherlands Trial Register, www.onderzoekmetmensen.nl/nl/trial/26952, NTR4457, *CINGLE* trial.

## 1 Introduction

Single-sided deafness (SSD) is a debilitating condition resulting in poor sound localization capabilities, reduced speech perception in noise, and decreased quality of life (QoL) (1–3). Next to the lack of binaural hearing, tinnitus is a prevalent and disabling symptom in a large majority of patients with SSD, which can further degrade their QoL (1, 4) and can also lead to psychological distress (5).

Although tinnitus is a common symptom, the exact pathophysiological mechanisms are not fully understood (6). Tinnitus is considered to be the consequence of changes in neural activity along the auditory pathway, including the auditory cortex, caused by reduced or lack of auditory input, typically due to local hair cell loss (7, 8). Therefore, hearing loss is considered the most important risk factor for tinnitus (9, 10).

For patients with SSD, the bone conduction device (BCD) and contralateral routing of sound hearing aids (CROS) are widely available treatment modalities. Although both devices show benefits in subjective speech perception and QoL (11–13), they do not stimulate the deprived auditory pathway of the impaired ear and are, therefore, not likely to reduce tinnitus (14, 15). In contrast, a cochlear implant (CI) provides input to the auditory nerve of the affected ear, thereby partially restoring the balance of excitation and inhibition along the auditory pathway and counteracting tinnitus origins (8, 16).

Several systematic reviews focused on the effect of CI on tinnitus in SSD patients (17–19). Overall, a clear reduction of tinnitus distress was found up to 72 months after cochlear implantation (17–19). However, these outcomes were all based on observational studies with a moderate to high risk of bias and mainly derived from studies with short-term outcomes. Although these results are promising, high level of evidence studies comparing different treatment options for SSD are needed to draw firmer conclusions on the tinnitus outcomes.

Therefore, the aim of the current study is to investigate the effect of a CI, BCD, CROS, and no treatment on tinnitus impact scores in a large sample size of SSD patients up to 24 months of follow-up. This study is part of an ongoing randomized controlled trial (RCT) investigating CI, BCD, and CROS, and no treatment for SSD patients (13, 20).

## 2 Patients and methods

### 2.1 Ethical considerations

This study is part of the CINGLE trial (Cochlear Implantation for single-sided deafness), an ongoing single-center RCT investigating CI, BCD, CROS, and no treatment for SSD patients. The research protocol of this study was approved by the Institutional Review Board of the University Medical Center Utrecht (NL45288.041.13) and is registered with the Netherlands Trial Register (http://www.onderzoekmetmensen.nl, NTR4457). For a detailed description of the CINGLE trial, we refer to the study protocol (13). Written informed consent was obtained from all participants between July 2014 and February 2019. This study reports data according to the CONSORT statement (21).

### 2.2 Study population and design

Adult (≥18 years) SSD patients were eligible for inclusion if they had a duration of deafness of a minimum of 3 months and a maximum of 10 years with a pure tone average hearing loss at 0.5, 1, 2, or 4 kHz equal to or more than 70 dB for the hearing-impaired ear and a maximum of 30 dB for the better ear. Patients had to be willing and able to participate in all scheduled procedures as outlined in the study protocol (13). Patients with retro cochlear pathology, abnormal cochlear anatomy, or an implanted BCD were excluded. There were no specific inclusion or exclusion criteria with regard to the participants' tinnitus complaints, which means that both patients with and without tinnitus could be included in the study.

**Figure 1:**
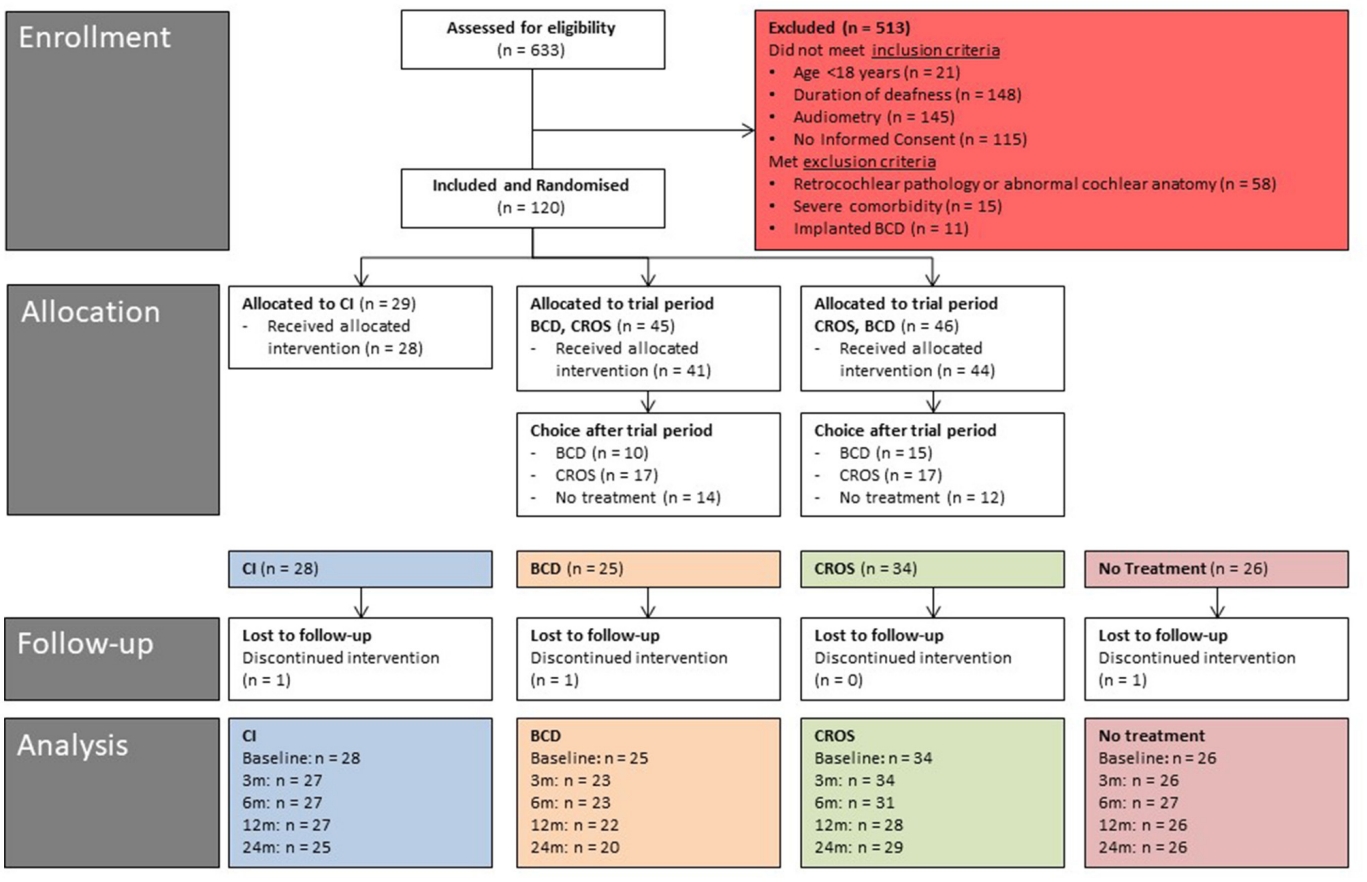
Study flow diagram (baseline - 24 months of follow-up). CI, cochlear implant; BCD, bone conduction device; and CROS, contralateral routing of sound hearing aid.

In total, 120 patients were randomized to one of three randomization groups by a web-based tool (ratio 2:3:3, block randomization): 29 patients to the CI group, 45 patients to the “first BCD, then CROS” trial period group, and 46 patients to the “first CROS, then BCD” trial period group. The study flow chart is presented in Figure 1.

If randomized to the CI group, implantation of a CI (Cochlear™ Nucleus^®^ CI422 Slim Straight or CI512 Contour Advance) was scheduled and performed by one of four experienced hearing implant surgeons according to a standardized protocol (mastoidectomy, tympanotomy, and round window insertion). Approximately 4 weeks after implantation, the CI was first activated, followed by rehabilitation. If randomized to one of the trial period groups, each device (BCD on headband, conventional CROS hearing aid) was tested for 6 weeks. After the BCD and CROS trial periods, patients indicated their choice of treatment: BCD, CROS, or no treatment. If a BCD was preferred, surgical implantation of the implant and abutment was scheduled with the fitting of the BCD (BAHA, Cochlear™ Baha^®^ BP110, or 5 Power.) 6 weeks later. If CROS was preferred, patients were fitted with a CROS hearing aid (Phonak Audeo Q50-312 or V50-312). Patients continued in follow-up unaided if neither treatment was preferred (i.e., the “no treatment” group).

In this study, we report on data from all four groups (CI, BCD, CROS, and no treatment) with regard to tinnitus outcomes at baseline (i.e., the unaided condition) and at 3, 6, 12, and 24 months of follow-up. For the CI, BCD, and CROS groups, the first follow-up measurement (3 months) took place 3 months after device activation/fitting. For the no treatment group, the first measurement occurred 3 months after the end of the trial periods.

### 2.3 Study procedures

#### 2.3.1 Tinnitus outcomes

Patients indicated whether they experienced tinnitus at baseline and at 3, 6, 12, and 24 months of follow-up. If answered yes, three different tinnitus questionnaires were completed (in Dutch). If they answered no, patients did not complete the tinnitus questionnaires and were excluded from the analysis.

##### 2.3.1.1 Tinnitus handicap inventory (THI)

The THI measures the impact of tinnitus on daily life and is a 25-item validated questionnaire with questions about tinnitus burden (22). Possible answers are “yes” (4 points), “sometimes” (2 points), and “no” (0 points). A higher score reflects a higher tinnitus impact on daily life and is graded as slight (0–16), mild (18–36), moderate (38–56), severe (58–76), or catastrophic (78–100). According to Zeman et al. (23), a THI score reduction of at least seven points is considered to be clinically relevant.

##### 2.3.1.2 Tinnitus questionnaire (TQ)

The TQ measures tinnitus-related distress. The TQ is a 52-item validated questionnaire to assess five dimensions of tinnitus complaints: emotional and cognitive distress, intrusiveness, auditory perceptual difficulties, sleep disturbances, and somatic complaints (24). Possible answers are “true” (2 points), “partly true” (1 point), and “not true” (0 points). A higher score reflects higher tinnitus distress (range 0–84 points). For this study, we used the validated Dutch version of the TQ (25). Tinnitus-related distress can be graded as light (0–30 points), moderate (31–46 points), severe (TQ 47–59 points), or very severe (60–84 points). The minimum change in the TQ score to be considered clinically relevant is set at a reduction of five points (26).

##### 2.3.1.3 Visual Analog Scale

The Visual Analog Scale (VAS) score was used to measure general tinnitus burden, represented by a straight line ranging from “no tinnitus burden” (rated as 0) to “maximum tinnitus burden” (rated as 100).

### 2.4 Hospital anxiety and depression scale

The Hospital Anxiety and Depression Scale (HADS) is a screening tool for anxiety and depression in non-psychiatric clinical populations (27, 28). The HADS is an important tool to evaluate the psychological burden of tinnitus. The degree of tinnitus burden is significantly influenced by psychological factors such as negative emotional reactions, cognitive misinterpretations, and avoidance behavior.

The HADS questionnaire contains 14 items: seven items each for the depression and anxiety subscales. Scoring for each item ranges from 0 to 3. A higher total score reflects a higher probability of anxiety or depression. The maximum score of each scale is 21 points: normal or no anxiety or depression (0–7), possible anxiety or depression (8–10), and probable anxiety or depression (11–21).

### 2.5 Follow-up

At baseline, all patients filled out all questionnaires at the clinic. At 3, 6, 12, and 24 months of follow-up, all questionnaires were filled out at home a few days prior to the follow-up at the clinic.

For the CI group, the VAS was completed for two different conditions (at 3, 6, 12, and 24 months of follow-up). One condition was when the CI was on (i.e., while using the CI, referred to as “CI-ON”), and the other was when the CI was off (i.e., while not using the CI, referred to as “CI-OFF”). For the “CI-OFF” condition, patients were asked to fill out the questionnaire based on their experience when the CI was turned off.

### 2.6 Statistical analysis

All analyses were conducted using SPSS version 25.0 (IBM Corp., Armonk, NY, USA). Kolmogorov–Smirnov and Shapiro–Wilk tests were used to determine the normality of the data. Continuous data are presented as mean (SD) or median [range]. Categorical data are presented as the total number. To compare patient and disease-specific characteristics and treatment groups at baseline, the chi-square test and one-way ANOVA were used in normally distributed data. Independent-Samples Kruskal-Wallis was used in data that were not normally distributed. Generalized estimating equations (GEE) were used to compare primary and secondary outcomes between groups at all follow-ups and between baseline and 24 months of follow-up for each group. Factors included in the GEE were “group” (0 = excluded, 1 = CI, 2 = BCD, 3 = CROS, 4 = no treatment) and “time” (0 =baseline, 1 = 3 months, 2 = 6 months, 3 = 12 months, 4 = 24 months). To correct for multiple testing, a Bonferroni correction was applied, i.e., the critical level of significance divided by the number of comparisons made (29). The critical level of significance is typically 0.05. The number of comparisons in our study was generally three [e.g., repeated measures for one study group vs. each other study group (*n* = 3)]. Hence, *p*-values of < 0.017 (0.05/3 = 0.017; two-sided) were considered to be statistically significant. Subject data were analyzed “as treated.”

## 3 Results

### 3.1 Patient characteristics

In total, 120 patients were randomized (Figure 1). Of the 29 patients randomized to the CI group, one patient was unwilling to undergo surgery and withdrew from the study before cochlear implantation. Hence, the CI group consisted of 28 patients after treatment allocation. Of the 91 patients randomized to the BCD and CROS trial period groups, seven patients did not start or complete the trial periods due to various reasons: six patients were lost to follow-up, and one patient continued in the no-treatment group (30). Notably, one of these seven patients rejected the test of the devices because of disabling tinnitus. One patient was implanted with a CI after negotiations with his insurance company. One patient could not complete the trial period due to health issues not related to SSD, one patient was disappointed by the randomization result, and three patients indicated a lack of motivation to complete the trial period for personal reasons. After the trial periods (*n* = 85), 25 patients chose BCD implantation, 34 patients chose CROS, and 26 patients chose to continue in the no-treatment group.

Table 1 shows all patients' patient characteristics and tinnitus outcomes after treatment allocation (i.e., the unaided situation). After treatment allocation, 105 of 113 (93%) patients indicated experiencing tinnitus, ranging from slight/light to catastrophic, in all treatment groups (according to THI and TQ scores). There were no significant differences between groups after treatment allocation regarding patient characteristics and tinnitus scores at baseline.

**Table 1:** Patient characteristics per allocated group.

	**Treatment group after allocation**				**Statistics**
	**CI**	**BCD**	**CROS**	**No treatment**	
	***n*** = **28**	***n*** = **25**	***n*** = **34**	***n*** = **26**	
**Gender**					
Male	13	10	21	9	ns^a^
Female	15	15	13	17	
**Age at inclusion (years)**					
Mean (SD)	52.5 (12.9)	56.0 (8.4)	52.1 (12.0)	51.5 (12.9)	ns^b^
**Deaf ear**					
Left ear	15	16	18	17	ns^a^
Right ear	13	9	16	9	
**Duration of deafness (years)**					
Median [range]	1.9 [0.3–10.0]	2.3 [0.3–10.0]	1.3 [0.3–10.0]	1.7 [0.3–10.0]	ns^c^
**PTA better ear (0.5–4 kHz) (dB)**					
Median [range]	15.0 [5.0–30.0]	12.5 [3.8–28.8]	16.3 [5.0–27.5]	15.6 [2.5–30.0]	ns^c^
**PTA poor ear (0.5–4 kHz) (dB)**					
Median [range]	96.3 [75.0–120.0]	92.5 [80.0–116.3]	93.8 [73.8–120.0]	93.8 [70.0–117.5]	ns^c^
**SSD etiology**					
Unknown	5	4	5	12	ns^a^
Iatrogenic	1	0	0	1	
Idiopathic sudden hearing loss	15	15	18	8	
Labyrinthitis	4	2	5	1	
Infection	0	0	2	1	
M. Meniere	3	3	3	1	
Traumatic	0	1	1	2	
**Presence of tinnitus**					
Yes	26	22	33	24	ns^a^
No	2	3	1	2	
**Tinnitus THI-score (0–100 points)**					
Median [range]	27 [2–74]	21 [0–84]	22 [0–88]	26 [0–88]	ns^c^
Slight (0–16)	10	10	11	4	ns^a^
Mild (18–36)	6	6	12	12	
Moderate (38–56)	6	4	7	6	
Severe (58–76)	4	1	2	0	
Catastrophic (78–100)	0	1	1	2	
**Tinnitus TQ-score (0–84 points)**					
Median [range]	26 [5–59]	24 [2–70]	21 [1–70]	30 [1–74]	ns^c^
Light (0–30)	15	15	22	13	ns^a^
Moderate (31–46)	6	2	7	5	
Severe (47–59)	5	3	3	4	
Catastrophic (60–84)	0	2	1	2	
**Tinnitus VAS-score (0–100 points)**					
Median [range]	70 [10–100]	70 [7–100]	50 [0–100]	51 [8–100]	ns^c^

CI, cochlear implant; BCD, bone conduction device; CROS, contralateral routing of sound hearing aid; PTA, pure tone average; SD, standard deviation; ns, not significant.

^a^Fisher's exact test.

^b^One-way ANOVA.

^c^Independent-samples Kruskal–Wallis test.

### 3.2 Device characteristics

In the CI group, the first 12 patients were implanted with a Nucleus^®^ CI422 with a slim straight electrode array. The latter 16 patients were implanted with a Nucleus^®^ CI512 with a contour advance electrode array. In the BCD group, the first three patients were fitted with a Baha^®^ BP110, and the remaining 22 patients were fitted with a Baha^®^ 5 Power.

### 3.3 Numbers of patients in analysis

Figure 1 shows the number of patients per treatment group at different follow-ups.

In the CI group (*n* = 28 patients after treatment allocation), one patient was lost to follow-up before the 3-month follow-up. This patient indicated that the follow-up measurements were too demanding. Eventually, this patient had the CI explanted 18 months after implantation because of unexplained pain complaints. Thus, 27 patients were analyzed at 3, 6, and 12 months. Between 12 and 24 months, two patients in the CI group wished to not use their CI anymore. In one of these patients, the CI was explanted 20 months after implantation because of unexplained pain complaints. As a result, 25 patients were analyzed at 24 months.

In the BCD group (*n* = 25 patients after treatment allocation), one patient decided not to undergo surgery and was lost to follow-up. One patient was implanted, but due to recurrent skin infections related to the BCD, the BCD was removed and was not re-implanted. This patient was further analyzed in the no-treatment group at 3, 6, 12, and 24 months. Therefore, 23 BCD patients were analyzed at 3 and 6 months. One patient became a non-user between 6 and 12 months and was further analyzed in the no-treatment group at 12 months. Thus, at 12 months, 22 BCD patients were analyzed. After 12 months, two patients became non-users. Hence, 20 patients were analyzed at 24 months.

In the CROS group (*n* = 34 patients after treatment allocation), two patients did not use their CROS anymore after the 3-month follow-up. These patients were measured without devices and analyzed in the no-treatment group at 6 months. Another patient was lost to follow-up after 3 months. Hence, 31 patients were analyzed at 6 months. After the 6-month follow-up, two other patients no longer used their CROS and were analyzed in the no-treatment group at 12 months. One patient was lost to follow-up after 6 months. Hence, 28 patients were analyzed at 12 months. After 12 months, two patients were lost to follow-up, and one patient from the no-treatment group purchased a CROS, which was measured and analyzed in the CROS group. Thus, at 24 months, 29 patients were analyzed in the CROS group.

In the no-treatment group (*n* = 26 patients after treatment allocation), one patient was lost to follow-up before the 3-month follow-up measurements. After 3 months, one patient was lost to follow-up. After 6 months of follow-up, four patients were lost to follow-up. As mentioned, one patient from the BCD group was analyzed in the no-treatment group at 3 and 6 months, and two patients from the CROS group were analyzed in the no-treatment group at 6 months. One patient from the no-treatment group was analyzed in the CROS group at 24 months.

### 3.4 Missing data

Outcomes of all questionnaires (THI, TQ, VAS, and HADS) were available for all patients at baseline. During follow-up, the percentage of missing data increased from 13% at 3 months to 20.5% at 24 months. On average, 12.7% of the data was missing for the THI and TQ. For the VAS, an average of 9.8% of data was missing. For the HADS, an average of 15% of the data was missing.

### 3.5 Tinnitus outcomes

Table 2 shows the number of patients with and without tinnitus per treatment group for each follow-up. At baseline, eight out of 113 patients (7%) indicated that they did not experience tinnitus. There were no patients who developed tinnitus after cochlear implantation or BCD implantation. In the CROS group, one patient did not have tinnitus at baseline but was reported to have tinnitus at 12 and 24 months of follow-up. No patients in the no-treatment group developed tinnitus during follow-up. Only in the CI group did the number of patients indicating that they were not experiencing tinnitus (anymore) increase from two out of 28 patients at baseline to seven out of 24 patients at 24 months. The number of patients reporting tinnitus in the BCD and CROS groups was stable during follow-up.

**Table 2:** Number of patients with and without tinnitus per group per follow-up.

**Tinnitus**	**CI**					**BCD**					**CROS**					**No treatment**				
	**Baseline**	**3 months**	**6 months**	**12 months**	**24 months**	**Baseline**	**3 months**	**6 months**	**12 months**	**24 months**	**Baseline**	**3 months**	**6 months**	**12 months**	**24 months**	**Baseline**	**3 months**	**6 months**	**12 months**	**24 months**
Yes	26	20	21	16	17	22	18	21	19	17	33	31	28	27	27	24	21	24	22	18
No	2	5	5	6	7	3	3	2	2	2	1	1	2	0	1	2	3	3	2	3
Missing	0	2	1	5	1	0	2	0	1	1	0	2	1	1	1	0	2	0	2	5
Total	28	27	27	27	25	25	23	23	22	20	34	34	31	28	29	26	26	27	26	26

CI, cochlear implant; BCD, bone conduction device; CROS, contralateral routing of sound hearing aid.

In the CI group; two patients did not have tinnitus at baseline. After cochlear implantation; three patients reported not having tinnitus anymore at 3 months. Later; one patient did not have tinnitus after 12 months; and another patient reported not having tinnitus after 24 months. No patients developed tinnitus after cochlear implantation.

In the BCD group; three patients did not have tinnitus at baseline. At 3; 6; 12; and 24 months; one of these patients switched to the no-treatment group. One patient reported having tinnitus at baseline and indicated not to have tinnitus anymore at a 3-month follow-up. However, the tinnitus returned after 6 months. No patients developed tinnitus after BCD implantation.

In the CROS group; one patient did not have tinnitus at baseline. However; this patient was reported to have tinnitus at 12 and 24 months. One patient with baseline tinnitus reported no longer having tinnitus at 6 months. However; the tinnitus returned at 12 and 24 months. One patient who had tinnitus at baseline at 3; 6; and 12 months reported not to have tinnitus anymore at 24 months.

In the no-treatment group; two patients did not have tinnitus at baseline. The patient from the BCD group without tinnitus who switched to the no-treatment group was analyzed in the no-treatment group at 3; 6; 12; and 24 months. At 12 months; one patient with no tinnitus at baseline is missing.

**Figure 2:**
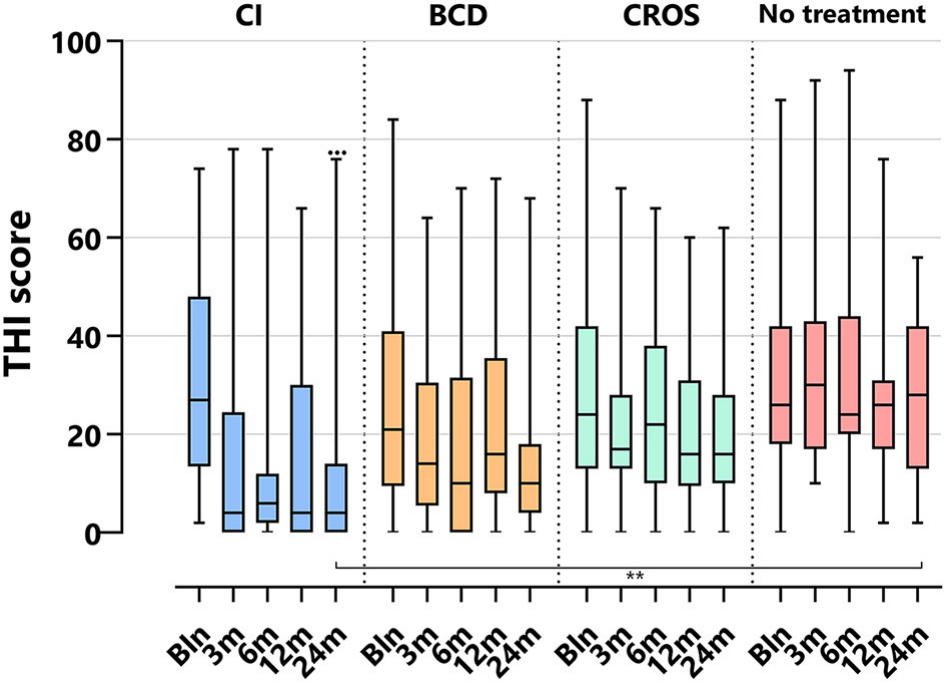
Tinnitus scores (THI) per treatment group and follow-up. THI, tinnitus handicap inventory; CI, cochlear implant; BCD, bone conduction device; and CROS, contralateral routing of sound hearing aid. The boxplots display the minimum; first quartile; median; third quartile; and maximum. After Bonferroni correction; *p*-values of < 0.017 (two-sided) were considered to be statistically significant. Statistical difference within the group; 24 months compared to baseline: ^•••^*p* < 0.001. Statistical difference between groups at 24 months of follow-up: **p < 0.017.

**Figure 3:**
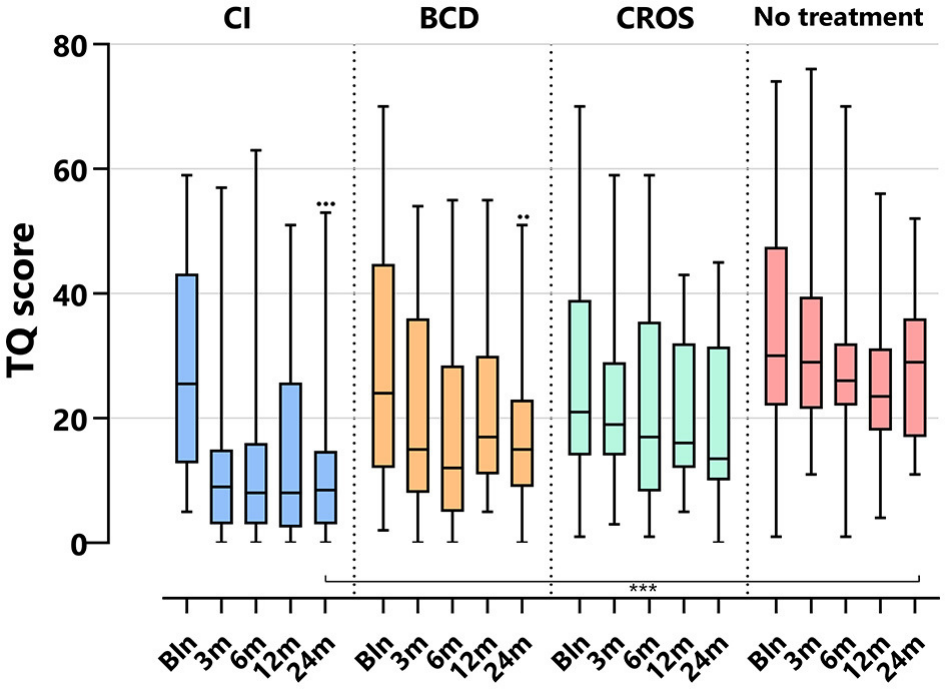
Tinnitus scores (TQ) per treatment group and follow-up. TQ, tinnitus questionnaire; CI, cochlear implant; BCD, bone conduction device; and CROS, contralateral routing of sound hearing aid. The boxplots display the minimum; first quartile; median; third quartile; and maximum. After Bonferroni correction; *p*-values of < 0.017 (two-sided) were considered to be statistically significant. Statistical difference within the group; 24 months compared to baseline: ^•••^*p* < 0.001; ^••^*p* < 0.017. Statistical difference between groups at 24 months of follow-up: ***p < 0.001.

**Figure 4:**
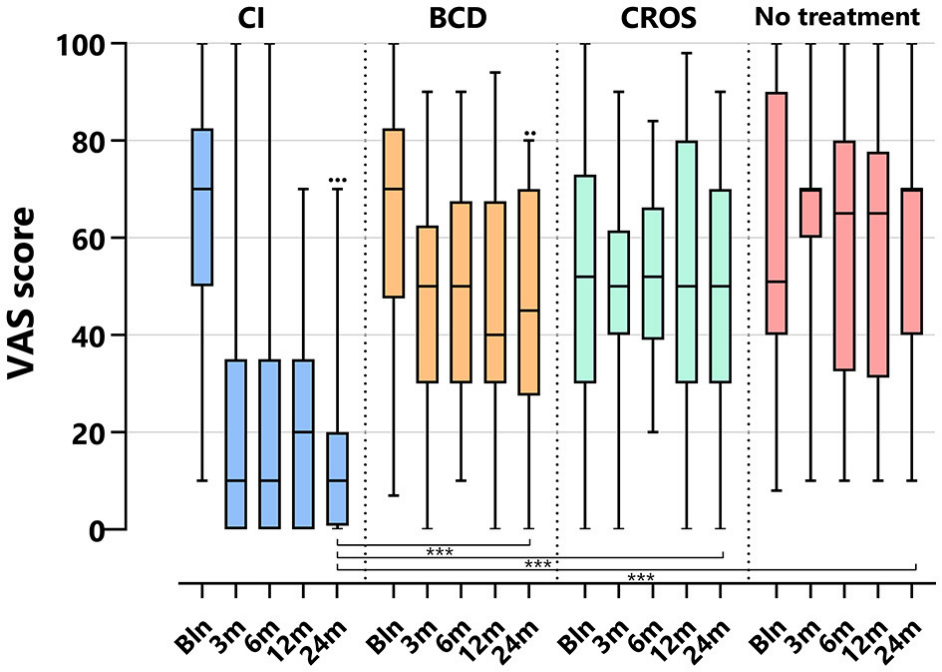
Tinnitus scores (VAS general tinnitus burden) per treatment group and follow-up. VAS, visual analog scale; CI, cochlear implant; BCD, bone conduction device; and CROS, contralateral routing of sound hearing aid. The boxplots display the minimum; first quartile; median; third quartile; and maximum. After Bonferroni correction; *p*-values < 0.017 (two-sided) were considered to be statistically significant. Statistical difference within the group; 24 months compared to baseline: ^•••^*p* < 0.001; ^••^*p* < 0.017. Statistical difference between groups at 24 months of follow-up: ***p < 0.001.

**Figure 5:**
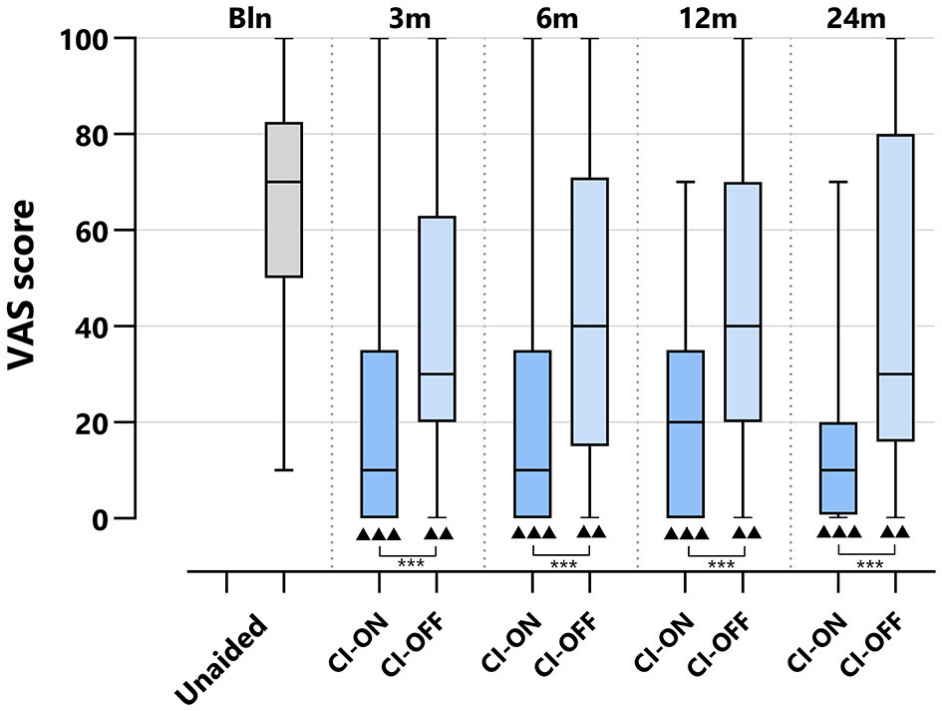
VAS scores of CI group per treatment group and follow-up: differences CI-ON vs. CI-OFF. VAS, Visual Analog Scale; CI, Cochlear Implant; CI-ON, condition in which the patient uses the CI; CI-OFF, condition in which the CI is off. The boxplots display the minimum; first quartile; median; third quartile; and maximum. After Bonferroni correction; *p*-values of < 0.017 (two-sided) were considered to be statistically significant. Statistical difference within the group; 24 months compared to baseline: ^▴▴▴^*p* < 0.001; ^▴▴^*p* < 0.017. Statistical difference between groups at the same follow-up: ***p < 0.001.

Table 3 and Figures 2–**5** show tinnitus outcomes.

**Table 3:** Tinnitus and HADS outcomes.

**Tinnitus and HADS outcomes**							
**Questionnaire**	**Median [range]**					**Difference**	***p*** **-value of difference**
	**Baseline**	**3 months**	**6 months**	**12 months**	**24 months**	**24 months–baseline**	**24 months–baseline**
**THI**							
CI	27 [2–74]	4 [0–78]	7 [0–78]	4 [0–66]	4 [0–76]	**−23**	**< 0.001**
BCD	21 [0–84]	14 [0–64]	10 [0–70]	16 [0–72]	12 [0–68]	−9	ns
CROS	22 [0–88]	18 [0–70]	23 [0–66]	16 [0–60]	16 [0–62]	−6	ns
No treatment	26 [0–88]	30 [10–92]	25 [0–94]	25 [0–76]	29 [2–68]	+3	ns
**TQ**							
CI	26 [5–59]	9 [0–57]	8 [0–63]	8 [0–51]	9 [0–53]	**−17**	**< 0.001**
BCD	24 [2–70]	15 [0–54]	12 [0–55]	17 [5–55]	15 [0–51]	**−9**	**0.002**
CROS	21 [0–70]	19 [3–59]	17 [1–59]	16 [5–43]	14 [0–45]	−7	ns
No treatment	30 [1–74]	29 [11–76]	26 [1–70]	24 [4–56]	29 [11–52]	−1	ns
**VAS**							
CI–ON	70 [10–100]	10 [0–100]	10 [0–100]	20 [0–70]	10 [0–70]	**−60**	**< 0.001**
CI–OFF	70 [10–100]	30 [0–100]	40 [0–100]	45 [1–100]	30 [0–95]	**−40**	**< 0.001**
BCD	70 [7–100]	50 [0–90]	50 [10–90]	40 [0–94]	45 [0–80]	**−25**	**0.004**
CROS	50 [0–100]	50 [0–90]	52 [20–84]	50 [0–98]	50 [0–90]	0	0.027
No treatment	51 [8–100]	70 [10–100]	65 [10–100]	65 [10–100]	70 [10–100]	+20	ns
**HADS anxiety**							
CI	5 [0–12]	3 [0–16]	1.5 [0–13]	1.5 [0–8]	3 [0–12]	**−2**	**0.006**
BCD	5.5 [0–14]	5 [0–8]	5 [0–15]	4 [0–9]	3 [0–13]	−2.5	ns
CROS	3.5 [0–17]	4 [0–11]	4 [0–15]	3 [0–13]	4 [0–17]	+0.5	ns
No treatment	4 [0–17]	2 [0–18]	3 [0–17]	2 [0–17]	3 [0–18]	−1	ns
**HADS depression**							
CI	7 [2–13]	6 [5–11]	6 [4–11]	6 [5–10]	6 [4–10]	**−1**	**0.005**
BCD	7 [5–13]	8 [5–14]	7 [4–13]	4 [4–15]	7 [2–12]	0	ns
CROS	7 [4–23]	6.5 [3–9]	7 [4–710]	6.5 [4–9]	6 [4–11]	−1	ns
No treatment	7 [5–12]	7 [5–11]	8 [5–14]	7 [5–9]	7 [4–11]	0	ns

CI, cochlear implant; BCD, bone conduction device; CROS, contralateral routing of sound hearing aid; THI, tinnitus handicap inventory; TQ, tinnitus questionnaire; VAS, visual analog scale; CI-ON, condition in which the patient is using the CI; CI-OFF, condition in which the CI is off.

Bold values indicate statistically significant differences. After Bonferroni correction; p-values < 0.017 (two-sided) were considered to be statistically significant.

#### 3.5.1 THI

Figure 2 depicts the THI scores per treatment group and follow-up. For the CI group, the THI score decreased significantly at 24 months compared to baseline [median baseline score of 27 (2–74) decreased to 4 (0–76) at 24 months (*p* < 0.001)]. For the BCD, CROS, and no-treatment groups, no statistically significant changes in tinnitus impact scores were observed at 24 months compared to baseline.

At 24 months, only the THI score for the CI (4 [0–76]) group was significantly lower than the THI score for the no-treatment group (29 [2–68], *p* < 0.017).

#### 3.5.2 TQ

Figure 3 depicts the TQ scores per treatment group and follow-up. For the CI group, the TQ score decreased significantly at 24 months compared to baseline [median baseline score of 26 (5–59) decreased to 9 (0–53) at 24 months (*p* < 0.001)]. For the BCD group, the TQ score decreased significantly at 24 months compared to baseline [median baseline score of 24 (2–70), decreased to 15 (0–51) at 24 (*p* < 0.017)]. No statistically significant change in tinnitus distress was observed at 24 months compared to baseline for the CROS and no-treatment groups.

At 24 months, only the TQ score for the CI (9 [0–53]) group was significantly lower than the TQ score for the no-treatment group (29 [11–52], *p* < 0.001).

#### 3.5.3 VAS

Figure 4 depicts the VAS tinnitus burden scores per group and follow-up. For the CI group, the VAS score decreased significantly at 24 months compared to baseline [median baseline score of 70 (10–100) decreased to 10 (0–70) at 24 months (*p* < 0.001)]. For the BCD group, the VAS score decreased significantly at 24 months compared to baseline [median baseline score of 70 (7–70) decreased to 45 (0–80) at 24 months (*p* < 0.017)]. No statistically significant changes in tinnitus impact were observed at 24 months compared to baseline for the CROS and no-treatment groups.

At 24 months, the VAS score for the CI group (10 [0–7]) was significantly lower than the VAS score for the BCD (45 [0–80]), CROS (50 [0–90], and no-treatment groups (70 [10–100], *p* < 0.001).

Figure 5 depicts the VAS scores for the CI-ON and CI-OFF configurations per follow-up.

For both the CI-ON and CI-OFF configurations, the VAS scores decreased significantly at all follow-ups compared to baseline: the median baseline score of 70 [10–100] decreased to 10 [0–70] for the CI-ON condition and to 30 [0–95] for the CI-OFF condition at 24 months of follow-up (*p* < 0.017). At all follow-ups, the VAS score for the CI-ON configuration was significantly lower than for the CI-OFF group (*p* < 0.001).

### 3.6 HADS

**Figure 6:**
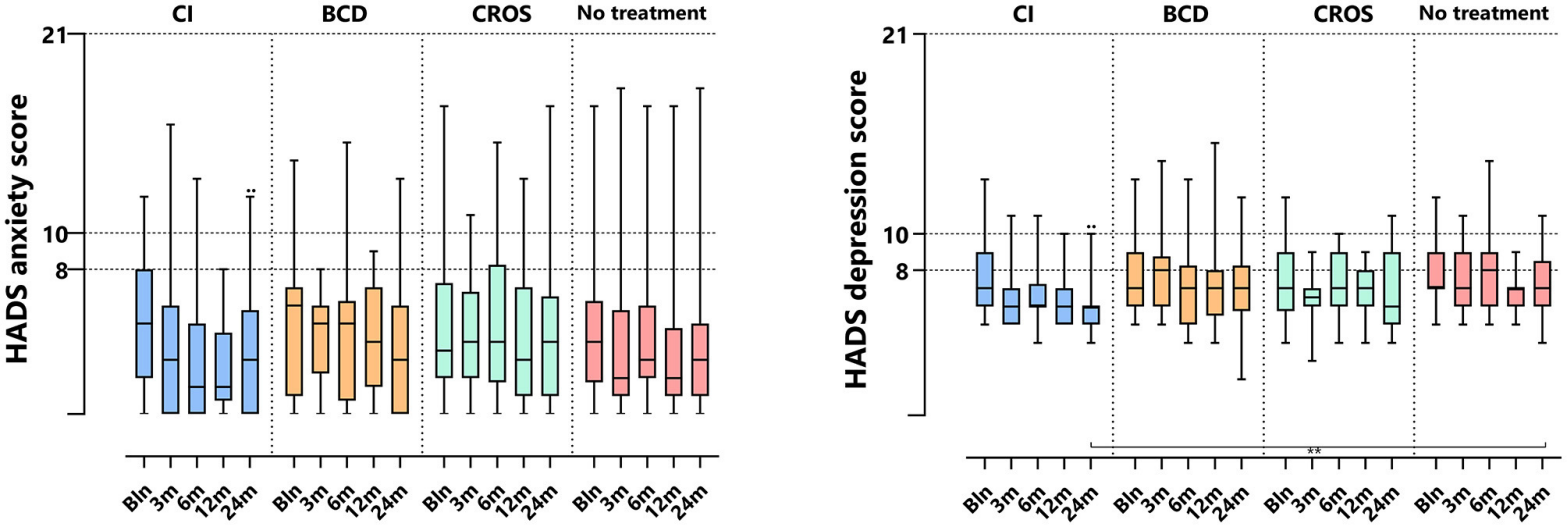
HADS anxiety and depression subscale scores per treatment group and follow-up. HADS, hospital anxiety and depression scale; CI, cochlear implant; BCD, bone conduction device; and CROS, contralateral routing of sound hearing aid. The boxplots display the minimum; first quartile; median; third quartile; and maximum. After Bonferroni correction; *p*-values of < 0.017 (two-sided) were considered to be statistically significant. Statistical difference within the group; 24 months compared to baseline: ^••^*p* < 0.017. Statistical difference between groups at 24 months of follow-up: **p < 0.017.

Table 2 and Figure 6 show HADS outcomes. Figure 6 depicts the HADS anxiety and depression scores per group and follow-up. For the HADS anxiety subscale, the median scores at baseline were 5 [0–12] for the CI group, 5.5 [0–14] for the BCD group, 3.5 [0–17] for the CROS group, and 4 [0–17] for the no-treatment group. Important to note is that although there is a wide range of scores, the median scores for all groups suggest no indication of anxiety at baseline. Only for the CI group, the anxiety score decreased significantly at 24 months compared to baseline [median baseline score of 5 (0–12) compared to 3 (0–12) at 24 months of follow-up (*p* < 0.017)]. For the CROS, BCD, and no-treatment groups, no statistically significant changes were observed in the anxiety subscale scores at 24 months compared to the baseline. There were no statistically significant differences between groups at 24 months of follow-up.

For the HADS depression subscale, the median scores at baseline were 7 [2–13] for the CI group, 7 [5–13] for the BCD group, 7 [4–23] for the CROS group, and 7 [5–12] for the no-treatment group. The median depression scores for all treatment groups indicate that there is no indication of depression at baseline. However, only the CI group showed a significant decrease in depression score at 24 months of follow-up, with a median score of 6 compared to the baseline median score of 7 [2–13] compared to 6 [4–10] at 24 (*p* < 0.017). No statistically significant changes were observed for the BCD, CROS, and no-treatment groups over the same period. At 24 months, the HADS depression score for the CI (6 [4–10]) group was significantly lower than for the no-treatment group (7 [4–11], *p* < 0.017).

### 3.7 Serious adverse events

There were three related serious adverse events (SAEs) during 2 years of follow-up: one participant in the BCD group had an implant extrusion and chose to be reimplanted. Two participants in the CI group experienced unexplainable pain and eventually had the CI surgically explanted (18 and 20 months after implantation).

There were also unrelated SAEs: one participant in the CI group had a transient ischemic attack several months after implantation; in the CROS group, one participant had a myocardial infarction for which he underwent percutaneous coronary intervention, and one participant underwent sinus surgery due to nasal polyps; in the no-treatment group, one participant had an arm fracture that required surgery, and one participant developed leukemia.

## 4 Discussion

### 4.1 Interpretation of results

In this RCT, the effect of CI, BCD, CROS, and no treatment on tinnitus outcomes in patients with SSD was investigated for up to 24 months of follow-up. The CI group showed the largest statistically significant decrease in all tinnitus impact scores up to 24 months of follow-up. The median THI score for the CI group decreased by 23 points, the TQ score decreased by 17 points, and the VAS score was 60 points at 24 months of follow-up compared to baseline. Moreover, at 24 months of follow-up, seven out of 24 (29 %) CI patients indicated experiencing complete resolution of tinnitus. No patients in the CI group reported the onset of tinnitus after cochlear implantation.

#### 4.1.1 Cochlear implantation

The beneficial tinnitus outcomes after cochlear implantation in SSD patients found in our RCT are in line with previous observational (cohort) studies in this field (17–19). The systematic review by Levy et al. (18) found that in 247 SSD patients, a CI resulted in a mean decrease of THI scores of 35.4 points (95% CI 55.8–15.0, *p* < 0.001). A weighted proportion of 14.9% of patients experienced complete resolution of tinnitus. The review by Idriss et al. included 31 observational studies investigating CI for SSD (3 up to 72-months follow-up) and concluded that for all included studies, CI reduced tinnitus significantly (evaluated using validated subjective tools). Moreover, the results followed a similar pattern in studies where tinnitus was assessed as a primary complaint or not (19). Our results are also comparable to the meta-analysis by Daher et al. (31), in which CI users reported a significant reduction in tinnitus severity as measured by the THI (mean difference, −29.97; 95% CI, −43.9 to −16.1; *p* < 0.001).

It is important to note that in our study, tinnitus was not assessed as a primary complaint. Our RCT was designed to investigate various treatment options for SSD patients with speech perception in noise as a primary outcome and tinnitus impact as one of the secondary outcomes. Participants in our study had, on average, a mild-to-moderate tinnitus impact score at baseline (Table 1). However, despite our study's modest baseline tinnitus impact scores (and thus smaller room for improvement), we did observe a clinically relevant decrease in tinnitus impact scores up to 24 months for the CI and BCD groups. This decrease after cochlear implantation is comparable to studies with higher tinnitus impact baseline values (i.e., studies where SSD patients were only included when SSD was accompanied by incapacitating tinnitus) (15, 32, 33).

In our study, tinnitus outcomes showed stable results throughout the 24 months of follow-up and already showed a clinically significant improvement 3 months after cochlear implantation. Such a short-term positive effect is to be expected based on the mechanism of action to electrically stimulate the auditory pathway to counteract tinnitus. The demonstrated stable improvement in tinnitus scores up to 24 months indicates that this (subjectively scored) improvement is not only explained by patients' optimism about receiving a CI and may discard a potential placebo effect. Parallel to this, in the review by Idriss et al. (19), eight of the included studies had a follow-up period of 24 months or more, including six prospective (1, 4, 32, 34–36) and two retrospective cohort studies (37, 38). Taking the methodological limitations of these studies into account, these studies also observed stable tinnitus reduction during their follow-up period.

Theoretically, for tinnitus, a beneficial effect is to be expected from electrical stimulation of the auditory nerve at the start of CI activation by immediately restoring the input to the auditory pathway. Following this theory, it makes sense that the effect had already been observed at the first follow-up at 3 months. Furthermore, the central auditory gain could be reduced with an associated tinnitus reduction after CI activation. Also, better awareness of environmental sounds could have a partial masking effect on tinnitus. To date, it is unclear how the duration of pre-implant deafness and the functional status of the auditory pathway of individuals will influence the extent of tinnitus improvement after cochlear implantation (16). To be noted, in our study, the maximum duration of deafness for the included patients was 10 years. Whether similar outcomes on tinnitus reduction can be expected in patients with a longer duration of pre-implant deafness is unclear and needs to be evaluated in future studies.

#### 4.1.2 CI ON/CI OFF

Notably, participants in our study rated their tinnitus experience (using the VAS score) in two conditions: one while using the CI (“CI-ON”) and the other while not using the CI (“CI-OFF”). In both conditions, a significant reduction in tinnitus was observed. However, the reduction was statistically significantly larger in the CI-ON condition than in the CI-OFF condition. Several other studies described this persistent tinnitus reduction, even when the CI was deactivated (1, 33). Whether the tinnitus reduction in the CI-OFF condition is the result of recall bias related to the subjective outcome measures used or can be attributed to physiological mechanisms related to the electrical stimulation, such as residual inhibition, needs to be explored. This could further facilitate effective tinnitus treatment and optimize electrical stimulation for relief.

#### 4.1.3 BCD, CROS, and no treatment

Next to the CI group, patients in the BCD group also showed a statistically significant decrease in tinnitus impact scores. The TQ scores decreased by 9 points at 24 months, and the VAS score decreased by 25 points compared to baseline. Although the decrease in scores for the BCD group is clearly less than for the CI group, we can conclude that this decrease is clinically relevant (23, 26).

As a BCD does not directly stimulate the deprived auditory pathway of the impaired ear, no direct beneficial effect on tinnitus would be expected. Studies on the effect of BCD on tinnitus outcomes in SSD patients are very scarce. In the retrospective cohort study by Lee et al. (39), mean THI scores before surgery (72.8 ± 16.1) had significantly improved by 6 months postoperatively (50.9 ± 18.9; *p* = 0.003) for the transcutaneous bone-conduction implant in SSD patients. In another cohort study, 2 of 18 SSD patients who completed the TQ mentioned that their tinnitus was less bothersome when wearing a BCD, whereas one patient reported a worsening of tinnitus while wearing it (40).

Reasons for the significant decrease in tinnitus impact scores observed after BCD implantation (and the non-significant decrease for the CROS group) are likely to be multifactorial and may be due to stimulation of residual cochlear function in the SSD ear (39, 41). Another explanation might be a placebo effect of BCD surgery, a placebo effect of wearing a hearing aid, or improved masking of tinnitus by both a BCD and CROS, as tinnitus is often described as more intrusive in silence and less profound in sound-enriched environments. Moreover, increased auditory input to the unaffected ear (both in BCD and CROS) may also decrease subjective tinnitus experience. Lastly, the general improvement of QoL and the improvement of speech perception in noise may also lead to a lower experienced tinnitus burden (13).

It is important to note that following the evolution of technologies (42), the use of even more powerful BCD devices (that reach higher frequencies and greater intensity) might show even more beneficial effects on tinnitus perception.

We did not observe a significant change in tinnitus impact scores for the no-treatment group. Spontaneous cessation of tinnitus has been studied previously in a systematic review and meta-analysis (43). The authors examined no-intervention or waiting-list tinnitus outcomes reported in trials in which participants with tinnitus on the active arm received different forms of tinnitus intervention. In contrast to our study, they found a statistically significant decrease in the impact of tinnitus over time (43).

### 4.2 Strengths and limitations

This study is part of an RCT investigating treatment options for SSD patients. It provides a high level of evidence on the tinnitus outcomes of different SSD treatments. The prospective nature had advantages, including standardized outcomes measurements and the reduction of missing data.

However, several limitations are to be considered. First, because BCD and CROS are part of standard clinical care for patients with SSD in the Netherlands, participants in the study were likely mostly motivated to receive a CI (not reimbursed in the Netherlands). This motivation could induce a positive attitude in the CI group, influencing subjective measures. One could argue that after some time, this positive attitude fades out. This was not seen in our study, in which tinnitus outcomes remained stable after 24 months of follow-up. Second, tinnitus outcomes are based on subjective scores when using questionnaires. Tinnitus loudness and pitch testing might provide additional informative measures when evaluating tinnitus outcomes. For future studies, we recommend the use of these measures. A third limitation is the use of different CI electrode arrays and BCD types during the study. The device variation is because we aimed to provide the highest standard of care during the study duration by evolving technologies. The use of different devices could have introduced a wider distribution of outcomes, though we expected this effect to be limited, and outcomes for these groups were considered univocal.

### 4.3 Future directions

In this study, we present the outcomes of an RCT evaluating the effect of a CI, BCD, CROS, or no treatment on tinnitus impact after 24 months of follow-up. This RCT is still ongoing, with a maximum duration of 5 years of follow-up. Other outcomes of sound localization, speech perception of noise, and quality of life will follow. Cost-utility analyses are needed to evaluate benefits and harms compared to societal costs, as cochlear implantation for SSD is not reimbursed in all countries. More and larger sampled studies are needed to compare our results to other implants for SSD (e.g., active middle ear implants, transcutaneous BCDs) and explain the influence of several patient and disease-related factors on tinnitus outcomes.

## 5 Conclusion

This RCT investigated the change in tinnitus impact scores in SSD patients with a CI, BCD, CROS, or no treatment up to 24 months of follow-up. Patients in the CI and BCD groups reported a significant reduction in their tinnitus impact scores, representing an overall improvement in tinnitus severity up to 24 months of follow-up. The CI group reported the largest clinically relevant reduction in tinnitus impact scores, a reduction that is reached after 3 months and remains stable up to 24 months of follow-up. CI appears to be superior to BCD and CROS, and no treatment to achieve partial or complete resolution of tinnitus in patients with SSD. Therefore, tinnitus might be considered an additional indication for cochlear implantation in SSD.

## Data availability statement

The raw data supporting the conclusions of this article will be made available by the authors, without undue reservation.

## Ethics statement

The studies involving humans were approved by Institutional Review Board of the University Medical Center Utrecht. The studies were conducted in accordance with the local legislation and institutional requirements. The participants provided their written informed consent to participate in this study.

## Author contributions

AW: Writing – review & editing, Writing – original draft, Visualization, Project administration, Investigation, Formal analysis, Data curation. KA: Writing – review & editing, Project administration, Investigation, Data curation. JH: Writing – review & editing, Project administration, Investigation, Data curation. JP: Writing – review & editing, Project administration, Methodology, Data curation, Conceptualization. WG: Writing – review & editing, Resources, Methodology, Funding acquisition, Conceptualization. RS: Writing – review & editing, Supervision, Resources. HV: Writing – review & editing, Supervision. AS: Writing – review & editing, Supervision, Resources, Methodology, Conceptualization.
